# Maintaining Clinical Training Continuity during COVID-19 Pandemic: Nursing Students’ Perceptions about Simulation-Based Learning

**DOI:** 10.3390/ijerph19042180

**Published:** 2022-02-15

**Authors:** Sitah Alshutwi, Fatmah Alsharif, Faygah Shibily, Almutairi Wedad M., Monir M. Almotairy, Maram Algabbashi

**Affiliations:** 1 College of Nursing, King Saud bin Abdulaziz University for Health Sciences, Riyadh 11481, Saudi Arabia; 2 King Abdullah International Medical Research Center (KAIMRC), Riyadh 14611, Saudi Arabia; 3 Medical Surgical Nursing Department, King Abdulaziz University, Jeddah 21589, Saudi Arabia; falsharif@kau.edu.sa; 4 Critical Care Nursing Department, King Abdulaziz University, Jeddah 21589, Saudi Arabia; fshibily@kau.edu.sa; 5 Maternity and Pediatric Nursing Department, King Abdulaziz University, Jeddah 21589, Saudi Arabia; walmutairi@kau.edu.sa; 6 Nursing Administration and Education Department, College of Nursing, King Saud University, Riyadh 12372, Saudi Arabia; malmotairy@ksu.edu.sa; 7 Nursing Sciences and Research Department, College of Nursing, Umm Alqura University, Makkah 24232, Saudi Arabia; mtghabbashi@uqu.edu.sa

**Keywords:** simulation-based learning, coronavirus diseases, nursing education

## Abstract

Background: Simulation-based learning (SBL) in nursing education is an innovative pedagogical approach that has significantly improved nursing education. Adopting SBL provides a controlled environment for meeting educational objectives without the risk of harm to real patients. Given that social distancing is required during the coronavirus disease (COVID-19) pandemic, SBL is a suitable alternative to clinical training for nursing students to learn and acquire the required clinical competencies. The study aimed to describe the effectiveness of SBL as a complete substitute for clinical experience from the perspective of students. This cross-sectional descriptive survey investigated students’ perceptions regarding the description of the effectiveness of SBL in four nursing colleges at four different universities across the Kingdom of Saudi Arabia. Settings: Four nursing colleges at four different universities across the Kingdom of Saudi Arabia. Participants included nursing students who attended simulation sessions. Data were collected by distributing a self-administrated online questionnaire, the Modified Simulation Effectiveness Tool (SET-M), which is a 19-item. Results: Approximately two-thirds of the participants were in their third (30.4%) and fourth (44.5%) academic year. The highest student presentation was for Site 1 (39.5%) and Site 2 (32.5%). Significant differences existed in all domains according to sex and university (*p* ≤ 0.001). There was a significant difference in relation to the level of agreement for pre-briefing, scenario, and debriefing domains (<0.001). Conclusions: SBL is a valuable teaching strategy that enhances nursing students’ self-awareness, self-confidence, clinical performance, and efficiency in performing procedures with considerable gender variation. Female students had more positive perceptions toward simulation effectiveness.

## 1. Introduction

Simulation-based learning (SBL) in nursing education is an innovative pedagogical approach that has significantly improved nursing education. Adopting SBL provides a controlled environment for meeting educational objectives without the risk of harm to real patients. Given that social distancing is required during the coronavirus disease (COVID-19) pandemic, SBL is a suitable alternative to clinical training for nursing students to learn and acquire the required clinical competencies. SBL in nursing education is an innovative pedagogical approach that has significantly improved nursing education. SBL is defined as “practicing realistic scenarios using a specialized manikin, computer software, or humans playing the role as the patient” [1]. Adopting SBL not only helps nursing students to mimic the real clinical experience, without exposing patients to any harm [2,3], but also helps students to develop knowledge, skills, and attitudes, with a sense of security for patients and for students [4] There is an increased reliance on the use of simulation due to limitations in clinical sites for student training, the lack of nursing instructors and faculty, and also due to the improved quality of nursing training provided through SBL [5]. Simulation helps in creating an environment that resembles the real environment in hospitals and supports students to gain nursing experiences, apply nursing skills, handle difficulties and concerns, and even make mistakes without causing any harm for patients, all in a safe environment [6,7].

SBL has become a trend in contemporary nursing education since it provides real clinical experience to students. The literature revealed different widespread implementation of simulation within the curriculum; in some programs simulation is used as part of clinical time using case scenarios, while other programs used simulation as a substitute for clinical training [8,9,10]. In Saudi Arabia, SBL is integrated in nursing programs at different levels, using low to high fidelity manikins, whether as a substitute or complementary to clinical training [10,11]. Numerous education programs apply SBL as a realistic and affordable teaching method to meet learning objectives [8,9,10,11,12,13]. Adopting SBL provides a controlled environment for meeting educational objectives without the risk of harm to real patients [14].

### 1.1. Objectives of the Study

In Saudi Arabia, there have only been a few single-center related studies. The majority of nursing simulation studies in Saudi Arabia focus on student satisfaction and self-confidence in simulation learning [15] and assessing simulation-based instructions to traditional teaching [16]. Therefore, this study aimed to describe the effectiveness of SBL as a complete substitute for clinical experience from the perspective of students. Moreover, this study aimed to explore the association between SBL effectiveness and students’ demographic characteristics.

### 1.2. Literature Review

Adopting SBL as a teaching modality has been recommended and supported by numerous nursing educational institutes, such as the National Council of State Boards of Nursing, which advocated for using simulation as a substitute for clinical training in all nursing courses [17]. Further, the gold standards for professional nursing education recommend using simulations in nursing education [18]. According to the NCSBN report, high-quality simulation practices will substitute for up to 50% of real clinical training in nursing programs [19,20].

Simulation allows nursing students to practice theory by connecting what they have learned in class to what they encounter in the hospital environment [21]. It has been shown that using simulation in nursing courses helps in achieving expected learning objectives [12], improving acquired knowledge [22] and enhancing learning satisfaction [23].

Many studies have evaluated the effectiveness of SBL in nursing education and found positive educational effects [24,25]. A meta-analysis study revealed that a medium-to-large effect size (0.70) suggested the effectiveness of adopting SBL in nursing education [25]. These findings were similar to the findings of a study conducted on health professional education that reported that simulation training had moderate to large effects [26]. Further, a longitudinal, randomized, and controlled study on the effectiveness of using SBL as a substitute for clinical training revealed that replacing clinical hours with simulation provided nursing students with relevant and rich clinical experiences [27]. A meta-analysis suggested that SBL has a strong educational impact, especially in terms of the technical skills and how students perform skills in simulation sessions [25]. SBL provides the opportunity to practice skills in a safe and nonthreatening environment that facilitates skills acquisition [28]. A quasi-experimental study on the clinical competence of nursing students in safe medication administration practices revealed that students who received simulation training on medication safety had significantly better performance than those who did not receive it [29]. Some studies evaluated the effectiveness of specific aspects of simulation, such as debriefing. As a core component of SBL, debriefing helps learners to meet the objectives and learning needs [30]. A systematic review reported that debriefing was the most significant effective factor achieved in simulation [31].

In addition to acquiring knowledge and skills, much research discusses the benefits of integrated SBL in nursing education [2,4,28,32,33]. Evans and coauthors reported a significant positive impact of SBL on students’ decision-making, teamwork, communication, confidence, and clinical experience [28,34]. Gore et al. (2011) showed that students with preclinical simulation experience before human patient contact had significantly lower anxiety scores than the controls [6]. Similarly, Karadag and coauthors reported that SBL reduced anxiety levels [35]. Other studies highlighted the role of SBL in promoting the critical thinking of students [32,33].

Worldwide, the COVID-19 pandemic has caused an educational sector crisis, with the health education program—where clinical training features prominently in teaching strategies—being among the most affected. Numerous health educational colleges and programs have replaced real clinical training with SBL to sustain and support the vital role of the profession in terms of both education and practice. [36,37]. The use of SBL in the nursing education program is not uncommon; moreover, it has been considered as an alternative for filling the gaps in skills teaching strategy. Given that social distancing is required during the coronavirus diseases (COVID-19) pandemic, SBL is a suitable alternative to the clinical training of nursing students to learn and acquire the required clinical competencies. Therefore, there is a need to maintain a positive perception of SBL among students to maximize advantages. Students’ positive perceptions are essential for successful SBL. Several studies have reported a high satisfaction level with SBL [38]. Contrastingly, another qualitative study reported that students were unsatisfied and had a negative perception of their experience with SBL [39]. Therefore, it is essential to develop research data about students’ perceptions of SBL.

## 2. Materials and Methods

### 2.1. Research Design

This cross-sectional descriptive survey investigated students’ perceptions regarding the effectiveness of SBL.

### 2.2. Setting

This study was conducted in four nursing colleges at four different universities across the Kingdom of Saudi Arabia. The first setting, the Faculty of Nursing at King Abdulaziz University, has one bachelor program for female students only. The second setting is the King Saud Bin Abdulaziz for Health Sciences, which has one bachelor program for male and female students with on average, 400 students graduating yearly. The third setting is the Faculty of Nursing at Um AlQura University, which has one bachelor program for male and female students and has an average of 150 graduates every year. The fourth setting is the College of Nursing at King Saud University, which has one bachelor program for male and female students, with an average of 300 graduates yearly.

### 2.3. Study Sampling and Sample Size

The study included nursing students involved in SBL who attended simulation sessions. We applied convenience sampling by sending invitation emails of electronic self-report questionnaires to 375 students who receiving SBL. The sample size was calculated through power analysis using a confidence interval of 95% and an alpha of 0.05. The estimated target sample size was 197 students.

### 2.4. Tools for Data Collection

We used the Modified Simulation Effectiveness Tool (SET-M), which is a 19-item tool developed in 2005, that includes the following three subscales with acceptable internal consistency: a 2-item pre-briefing subscale (α = 0.833), a 12-item scenario subscale (α = 0.913), and a 5-item debriefing subscale (α = 0.908). The SET-M was used for nursing students as a valid and reliable tool with overall internal consistency (α = 0.936) [40]. A higher score of the tool reflects students’ favorable perceptions of the simulation.

### 2.5. Ethical Considerations

Approvals from the Institutional Review Boards were obtained from the study sites (IRBC/0283/21) on 14 February 2021. Participation in this study was voluntary and informed consent was obtained from participants. All participates were informed that their participation will not affect their academic performance. No identification information was obtained from participants.

### 2.6. Data Analysis

Statistical analyses were performed using Statistical Package for Social Sciences software package (SPSS Inc., Chicago, IL, USA) version 27. Descriptive statistics were implemented for demographic characteristics and perceptions of nursing students on experience variables of SBL effectiveness. Categorical variables are expressed as a number and percentage. Continuous variables are expressed as the range, mean, and standard deviation. The chi-square test was used to determine significant differences regarding the agreement level between categories of demographic characteristics. Analysis of variance and a t-test were used to determine the association of the demographic characteristics and nursing students’ perceptions regarding the SBL experience. Statistical significance was set at *p* ≤ 0.05.

## 3. Results

This study included 375 nursing students, the majority of whom were female (82%). The participants’ ages ranged between 19 and 26 years old (22 ± 1.22), and the average mean of students’ GPA was 82.234 ± 11.159%. Approximately two-thirds of the participants were in their third (30.4%) and fourth (44.5%) academic years. The highest student participation was from Site 1 (39.5%) and Site 2 (32.5%) (Table 1).

Moreover, this study used the SET-M tool, which has three major subscales: the pre-briefing subscale (2-item), scenario subscale (12-item), and debriefing subscale (3-item). The analysis showed that there were significant differences in pre-briefing, scenario, and debriefing items among participants (Table 2). In the pre-briefing items, over half of the participants strongly agreed that the pre-briefing increased their confidence (*p* < 0.001, 55%) and benefited their learning experience (*p* < 0.001, 65%). In the scenario items, there were significant results in all 12 items of the subscale (*p* < 0.001). More than half of the students strongly agreed that the scenario prepared them to better respond to changes in patient condition (*p* < 0.001, 53%) and increased their confidence in their nursing assessment skills (*p* < 0.001, 52%), in prioritizing skills (*p*, 0.001, 55%), in communicating skills with patients (*p*, 0.001, 58%), in reporting information to the healthcare team (*p*, 0.001, 53%), and in providing interventions that foster patient safety (*p* < 0.001, 52%) (Table 2). Similarly, there were significant results in all debriefing subscale items among students (*p* < 0.001). Over half strongly agreed that the debriefing section of the simulation has contributed to their learning experience (*p* < 0.001, 52%) and allowed them to verbalize their feelings before focusing on the scenario (*p* < 0.001, 53%). Finally, the students strongly agreed that debriefing of the simulation helped them improve their clinical judgement (*p* < 0.001, 58%) and provided them opportunities to self-reflect on their performance during the simulation (*p* < 0.001, 56%) (Table 2).

Furthermore, the overall levels of agreement for the pre-briefing, scenario, and debriefing domains were 67.7%, 55.5%, and 53.3%, respectively (Table 3). There was a high overall level of agreement (52.5%). Also, there was a significant difference in relation to the level of agreement for the pre-briefing, scenario, and debriefing domains (Table 2, Table 3 and Table 4) (*p* < 0.001).

This study also tested the difference between the tool subdomain scores and demographic data, which were age, gender, GPA, academic years, and university site (Table 4). In the pre-briefing subdomain, it was found that there was a significant difference in response among students’ genders (*p* < 0.001) and university sites (*p* < 0.001). Female participants reported a higher mean (3.33 ± 0.907) than their male counterparts in the total subscore for pre-briefing. Similarly, there were significant differences between the scenarios’ total subscore and genders and university sites (*p* < 0.001). Female students reported a higher scenario subscore (19.24 ± 5.17) than male students (12.64 ± 4.74). University Site 1 showed the highest scenario subscore (19.85 ± 4.19) among other sites. Also, the study showed a significant difference between genders and university sites in the debriefing total subscore (*p* < 0.001). Female participants reported higher debriefing subscores than male students (7.85 ± 2.32, 6 ± 1.93, respectively). In addition, University Site 1 (8.52 ± 1.92) showed the highest debriefing subscore of all sites. Lastly, gender and university sites showed significant differences among participants in the total SET-M tool score. Female students (29.42 ± 7.74) showed a higher total score in the tool than their male counterparts (21.06 ± 6.78). Also, University Site 1 showed the highest total score of all sites participating in this study.

Finally, this study tested the relationship between subscores and the total score of the SET-M tool and two variables—age and GPA. There was a significant relationship between the debriefing subscore and student GPA (*p* = 0.022) (Table 5).

## 4. Discussion

Medical and nursing training in the modern era are multimodular; moreover, SBL could be crucially involved in refining training standards [41,42]. More than half of the students had a favorable perception regarding SBL, which indicated a bright prospect regarding its acceptance if implemented into the training module. In our study, there were higher agreement levels among women and Site 1 students. Additionally, we found a global positive and the greatest perception regarding SBL in all domains, which significantly differed according to sex and university. Specifically, there was a significantly higher favorable perception toward SBL among women and Site 1 students. Consistently, Albagawi and colleagues (2021) have found significantly more favorable outcomes—such as student satisfaction and self-confidence—of simulation-based learning among female students than male students [15]. In contrast, a previous study in Saudi Arabia by Mohamed and Fashafsheh (2019) found that male students have significantly more favorable perceptions of simulation-based training in terms of their clinical competencies. Thus, further research is needed to explore how perceptions of SBL may differ based on gender [43].

Most participants agreed that pre-briefing improved their learning and self-confidence. Furthermore, we found that students considered debriefing as beneficial to their learning, as it allows them to verbalize their feelings, improve clinical judgment, improve self-reflection, and build a constructive evaluation. Our findings are consistent with those of the study conducted by Saied, who reported that human simulation is an effective teaching and learning modality for pediatric nursing students. This study could facilitate the elucidation of how SBL may affect students’ knowledge, self-efficacy, and confidence [44].

Our findings are consistent with those of Presado and Alamrani [16,45] who reported that SBL with high fidelity is essential for nurse training, which reinforces the existing pedagogical practice in the studied context. Our participants considered that SBL could facilitate the development of competencies; moreover, our findings demonstrated that the different scenario subdomains contributed to the competencies in different domains. The scenario domain involves professional, ethical, and legal responsibility, as well as the provision and management of care. Haukedal and coauthors assessed the impact of new pedagogical interventions with respect to knowledge acquisition in SBL among nursing students and concluded that SBL improved knowledge levels regarding the disease and pathophysiology [46].

Our findings suggest that it may be easier to acquire knowledge regarding symptoms and actions through visualization, i.e., by managing the actual symptoms of deteriorating patients, watching themselves on videos, or experiencing specific scenarios. Sarfati and coauthors reported that properly regulated simulation could facilitate the training of staff for both exceptional and standard events [47]. By integrating human factors, a well-designed simulation program can effectively prevent iatrogenic risk related to medication errors. Lee and Park assessed SBL from a different perspective and investigated differences in the perception of SBL between nursing students and instructors [48]. Consistent with our findings, they reported that students prioritized learning outcomes and that SBL improved nursing skills. Additionally, consistent with our findings, El-Gebaly and coauthors focused on the effect of a reflective debriefing strategy on nursing education and reported that a reflective debriefing strategy had a positive influence on the clinical performance and efficacy, observation, evaluation, critical thinking skills, awareness, and thought processes of nursing students [49].

We found that the simulation effectiveness tool significantly varied according to demographic characteristics. Compared with male students, female students reported significantly higher simulation effectiveness tool scores. Furthermore, there were significant between-site differences in simulation effectiveness scores; moreover, there was a significant positive correlation of GPA with the debriefing domain. To our knowledge, there have been very limited previous studies on the differences in students’ perceptions of simulation effectiveness based on their demographic characteristics. However, there have been other studies on the relevant outcomes of simulation, including students’ performance, self-confidence, and managing deteriorating patients, with inconsistent previous findings. For example, students with more years of experience had better scores in managing deteriorating patients [50]; additionally, self-confidence negatively correlated with age [51]. Nevertheless, there was no significant relationship of students’ satisfaction of SBL with students’ demographic characteristics [51]. Therefore, our findings warrant further studies on the role of sex and university sites in students’ perceptions of simulation effectiveness, with comparable sample sizes of female and male participants and study sites.

## 5. Conclusions

SBL is a valuable teaching strategy that can enhances nursing students’ learning outcomes. Moreover, SBL can help students to develop their evaluation, critical thinking, observation, and communication skills. Additionally, it allowed students to independently master the procedure and identify their own learning needs. In this study, students provided positive feedback regarding all of the SBL domains (pre-briefing, scenario, and debriefing).

Several recommendations for research and practice emanated from this study. There is a need for further research on how students’ perceptions of simulation effectiveness vary; this can be analyzed using studies with comparable sample sizes. Future studies may include the characteristics of the study sites with respect to simulation preparedness (simulation fidelity, dose/integration into learning, scenario preparation toward course learning outcomes, and the number of faculties prepared to use simulation learning). Additionally, future studies may employ advanced statistical approaches with adjustment for differences and complexities of contributing factors—including universities, faculties, and student characteristics—as well as the possible interactions between these factors.

Nursing schools may consider evaluating the integration of SBL into their curriculums to foster students’ learning. This integration could involve ensuring that their faculty have adequate training in using equipment and manikins, designing learning scenarios, and evaluating the accomplishment of course outcomes through SBL. SBL integration should be aligned with training at clinical sites where it can help students provide safe and effective nursing care. Additionally, comprehensively evaluating the overall practical experience at simulation laboratories and clinical sites can facilitate alignment between these two training components and help students to excel in their learning journey in prelicensure programs.

## Figures and Tables

**Table 1:** The frequency and percentage regarding demographic data (Gender, Age, GPA, Academic year, University) in study group (*n* = 375).

Demographic Data		*N*	%
Gender	Female	308	82.1
	Male	67	17.9
Age	Range	19–26	
	Mean ± SD	21.697 ± 1.215	
GPA%	Range	54.6–98.8	
	Mean ± SD	82.234 ± 11.159	
Academic year	Second	48	12.8
	Third year	114	30.4
	Fourth year	167	44.5
	Graduated	46	12.3
University	Site 1	148	39.5
	Site 2	122	32.5
	Site 3	63	16.8
	Site 4	42	11.2

**Table 2:** The frequencies and percentages of agreement for the points of the domains (*n* = 375).

			Do Not Agree		Somewhat Agree		Strongly Agree		Chi-Square	
			*N*	%	*N*	%	*N*	%	X^2^	*p*-Value
Pre-briefing	1	Pre-briefing increased my confidence	7	1.9	161	42.9	207	55.2	175.6	<0.001 *
	2	Pre-briefing was beneficial to my learning	5	1.3	126	33.6	244	65.1	228.5	<0.001 *
Scenario	1	I am better prepared to respond to changes in my patient’s condition	37	9.9	139	37.1	199	53.1	107.3	<0.001 *
	2	I developed a better understanding of the pathophysiology	29	7.7	160	42.7	186	49.6	113.3	<0.001 *
	3	I am more confident of my nursing assessment skills	19	5.1	160	42.7	196	52.3	140.0	<0.001 *
	4	I felt empowered to make clinical decisions	24	6.4	182	48.5	169	45.1	123.1	<0.001 *
	5	I developed a better understanding of medications. (Leave blank if no medications in scenario)	51	13.6	169	45.1	155	41.3	66.5	<0.001 *
	6	I had the opportunity to practice my clinical decision making skills	32	8.5	158	42.1	185	49.3	106.7	<0.001*
	7	I am more confident in my ability to prioritize care and interventions	15	4.0	154	41.1	206	54.9	156.0	<0.001 *
	8	I am more confident in communicating with my patients	27	7.2	129	34.4	219	58.4	147.6	<0.001 *
	9	I am more confident in my ability to teach patients about their illness and interventions	24	6.4	133	35.5	218	58.1	151.3	<0.001 *
	10	I am more confident in my ability to report information to health care team	29	7.7	146	38.9	200	53.3	122.3	<0.001 *
	11	I am more confident in providing interventions that foster patient safety	15	4.0	165	44.0	195	52.0	148.8	<0.001 *
	12	I am more confident in using evidence-based practice to provide nursing care	24	6.4	189	50.4	162	43.2	125.3	<0.001 *
Debriefing	1	Debriefing contributed to my learning	10	2.7	170	45.3	195	52.0	161.2	<0.001 *
	2	Debriefing allowed me to verbalize my feelings before focusing on the scenario	18	4.8	155	41.3	202	53.9	146.2	<0.001 *
	3	Debriefing was valuable in helping me improve my clinical judgment	11	2.9	148	39.5	216	57.6	174.4	<0.001 *
	4	Debriefing provided opportunities to self-reflect on my performance during simulation	10	2.7	155	41.3	210	56.0	170.8	<0.001 *
	5	Debriefing was a constructive evaluation of the simulation	16	4.3	173	46.1	186	49.6	143.3	<0.001 *

* Significant result (two tailed, *p*-value < 0.05).

**Table 3:** Distribution of the level of all domains in study group (*n* = 375).

	Weak		Average		High		Overall Score	
	*N*	%	*N*	%	*N*	%	Range	Mean ± SD
Pre-briefing	10	2.7	111	29.6	254	67.7	0–4	3.171 ± 0.963
Scenario	64	17.1	103	27.5	208	55.5	1–24.	17.237 ± 5.523
Debriefing	20	5.3	155	41.3	200	53.3	0–10.	7.517 ± 2.360
Total	54	14.4	124	33.1	197	52.5	1–38.	27.925 ± 8.217

**Table 4:** The relation between domains and demographic data (Age, Gender, GPA, Academic year, University) study group (*n* = 375).

Domain	Demographic Data		*N*	Mean	±	SD	F or T	ANOVA or T-Test	
								Test Value	*p*-Value
Pre-briefing	Gender	Female	308	3.334	±	0.907	t	7.570	<0.001 *
		Male	67	2.418	±	0.855			
	Academic year	Second	48	3.042	±	0.988	f	2.494	0.060
		Third year	114	3.009	±	0.917			
		Fourth year	167	3.269	±	0.978			
		Graduated	46	3.348	±	0.948			
	University	Site 1	148	3.500	±	0.787	f	30.296	<0.001 *
		Site 2	122	3.352	±	0.899			
		Site 3	63	2.540	±	0.981			
		Site 4	42	2.429	±	0.859			
Scenario	Gender	Female	308	18.237	±	5.169	t	8.145	<0.001 *
		Male	67	12.642	±	4.741			
	Academic year	Second	48	16.375	±	5.782	f	0.913	0.434
		Third year	114	16.877	±	5.383			
		Fourth year	167	17.665	±	5.366			
		Graduated	46	17.478	±	6.145			
	University	Site 1	148	19.581	±	4.188	f	38.418	<0.001 *
		Site 2	122	17.943	±	5.197			
		Site 3	63	13.651	±	5.737			
		Site 4	42	12.310	±	4.464			
Debriefing	Gender	Female	308	7.847	±	2.317	t	6.079	<0.001 *
		Male	67	6.000	±	1.938			
	Academic year	Second	48	7.417	±	2.181	f	0.892	0.445
		Third year	114	7.465	±	2.219			
		Fourth year	167	7.701	±	2.291			
		Graduated	46	7.087	±	3.046			
	University	Site 1	148	8.520	±	1.915	f	25.023	<0.001 *
		Site 2	122	7.451	±	2.559			
		Site 3	63	6.476	±	2.109			
		Site 4	42	5.738	±	1.754			
Total	Gender	Female	308	29.419	±	7.737	t	8.185	<0.001 *
		Male	67	21.060	±	6.778			
	Academic year	Second	48	26.833	±	8.311	f	0.882	0.450
		Third year	114	27.351	±	8.044			
		Fourth year	167	28.635	±	7.935			
		Graduated	46	27.913	±	9.503			
	University	Site 1	148	31.601	±	6.122	f	39.853	<0.001 *
		Site 2	122	28.746	±	8.022			
		Site 3	63	22.667	±	8.289			
		Site 4	42	20.476	±	6.134			

* Significant result (two tailed, *p*-value < 0.05)

**Table 5:** The correlation between Age, GPA% and all Domains (*n* = 375).

	Age		GPA%	
	R	*p*-Value	R	*p*-Value
**Pre-briefing**	0.096	0.076	−0.045	0.437
**Scenario**	0.046	0.395	0.025	0.662
**Debriefing**	−0.048	0.378	0.133	0.022
**Total**	0.028	0.601	0.052	0.375
